# Mitophagy bridges glucose metabolism, inflammation and neuroprotection in astrocytes

**DOI:** 10.1080/15548627.2026.2623987

**Published:** 2026-02-12

**Authors:** Hanna Hakansson, Jack H Howden, Josef T Kittler

**Affiliations:** Neuroscience, Physiology and Pharmacology, University College London, London, UK

**Keywords:** Astrocyte, mitochondria, mitophagy, neurodegeneration, parkin, HK1, PINK1

## Abstract

Mitochondria regulate ATP production, calcium buffering, and apoptotic signaling, and clearing dysfunctional mitochondria by mitophagy is essential for cellular homeostasis. While PINK1-dependent mitophagy is well-characterized in neurons, its function in glial cells such as astrocytes is less understood. Our study demonstrates that PINK1-mitophagy in astrocytes occurs faster and with less spatial restriction compared to neurons. This pathway was specifically regulated in astrocytes by the glycolytic enzyme, HK2 (hexokinase 2), which forms a glucose-dependent complex with PINK1 following mitochondrial damage. Inflammation also induces HK2-PINK1 mitophagy, and its activation in astrocytes protects against cytokine-induced neuronal death. Our findings characterize a novel HK2-PINK1 pathway in astrocytes that bridges mitophagy, metabolism, and immune signaling.

**Abbreviation**: HK2: hexokinase 2; PD: Parkinson disease; PINK1: PTEN induced kinase 1; S65: serine 65

## Main text

**Figure 1.**
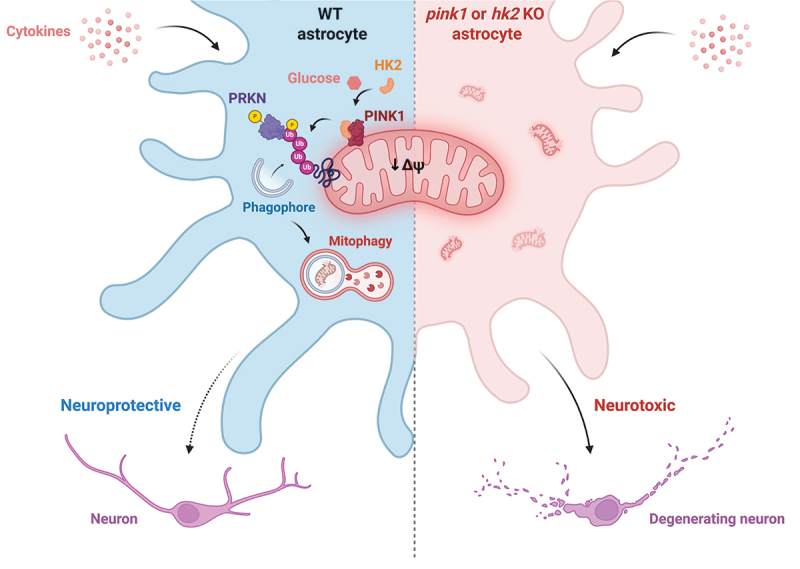
HK2-PINK1 mitophagy in astrocytes protects neurons against inflammation-induced death. HK2 forms a glucose-dependent complex with PINK1 in astrocytes to initiate mitophagy upon mitochondrial depolarization. Activation of this pathway is involved in astrocytes conferring neuroprotection against pro-inflammatory stimuli. Upon *hk2* or *pink1* knockout (KO) in astrocytes, mitophagy induction is lost, and so is the ability for these cells to protect neurons against inflammation. Diagram created with BioRender.

Canonical mitophagy begins with the accumulation of PINK1 (PTEN induced kinase 1) on damaged mitochondria, where it phosphorylates ubiquitin and the E3 ubiquitin ligase PRKN/parkin at serine 65 (S65). Phosphorylation of ubiquitin and PRKN triggers PRKN’s ligase activity, resulting in a feedforward loop of mitochondrial protein ubiquitination that targets damaged mitochondria for lysosomal degradation. Mitochondrial turnover is particularly important in cells with high energetic demands, such as neurons, with impaired mitophagy linked to progressive dopaminergic neuron loss in Parkinson disease (PD). While glial cells, including astrocytes and microglia, are increasingly recognized as key modulators of neurodegeneration, the mechanisms of mitophagy in these cells remain poorly understood. Our recent study [1] aimed to characterize PINK1-PRKN mitophagy in astrocytes and its role in neuroprotection (Figure 1).

Using mixed astrocyte-neuron cultures, we characterize two key adaptations of mitophagy in astrocytes. First, astrocytes initiate mitophagy more rapidly. Following mitochondrial depolarization by valinomycin, these cells show earlier S65 ubiquitin phosphorylation and translocation of fluorescent protein-tagged PRKN to mitochondria. Second, astrocytes exhibit less spatially restricted mitophagy. Whereas phosphorylated ubiquitin accumulates mainly in the soma of neurons, it is distributed throughout the soma and processes of astrocytes. As PRKN translocation and ubiquitin phosphorylation indicate mitophagy initiation, we used the pH-sensitive reporter mito-SRAI to confirm mitochondrial delivery to lysosomes. Astrocytes show increased mitolysosome formation, demonstrating a greater capacity to clear damaged mitochondria. These adaptations may enable astrocytes to maintain their bioenergetic demands while also supporting neuronal function through their proximity to synapses.

Loss of PINK1 impairs mitochondrial clearance in both astrocytes and neurons, suggesting that the faster, less spatially restricted mitophagy in astrocytes involves additional regulatory mechanisms. In a bioinformatics approach integrating mitophagy CRISPR screens with astrocyte and neuron transcriptomes, we identify HK2 (hexokinase 2), a mitochondrially localized enzyme involved in glucose metabolism, as a glia-specific mitophagy regulator. Astrocytes express high levels of HK2, and silencing HK2 blunts S65 ubiquitin phosphorylation and mitolysosome formation, confirming its functional role in mitophagy. HK2 has been reported to form a complex with PINK1, and co-immunoprecipitation reveals that this interaction occurs in astrocytes. Given HK2’s involvement in glycolysis, we tested HK2-PINK1 interaction under different metabolic states and found that a non-metabolizable glucose analog destabilizes the complex and impairs mitophagy. Unlike neurons, astrocytes are metabolically flexible, shifting between glycolysis and mitochondrial oxidative phosphorylation in response to substrate availability. This glucose-dependent stabilization of the HK2-PINK1 complex effectively links astrocyte mitophagy to metabolism, potentially enabling adaptive mitochondrial quality control under conditions of metabolic stress.

Damaged mitochondria can trigger inflammatory signaling in astrocytes, driving them toward neuroinflammatory and neurotoxic phenotypes that contribute to neurodegeneration. We show that exposure to pro-inflammatory cytokines enhances mitophagy in astrocytes by increasing S65 ubiquitin phosphorylation, particularly during the early phases of acute inflammation. This mitophagy induction is dependent on HK2-PINK1, as silencing either factor decreases S65 ubiquitin phosphorylation and mitolysosome formation. Interestingly, silencing HK2 or PINK1 in astrocytes increases cytokine-induced neuronal death in mixed cultures. Our results suggest a dual role for HK2-PINK1 in astrocytes: clearing damaged mitochondria to preserve astrocyte function and protecting neurons from inflammatory stress.

These findings have important implications for PD, where mutations in PINK1 and PRKN in familial forms of disease suggest defects in mitochondrial quality control in the underlying pathology. Although therapeutic strategies have focused on neurons, our study highlights astrocytic mitophagy as a previously underappreciated contributor to neuronal health. Neuroinflammation exacerbates neurodegeneration in PD, and the observation that HK2-PINK1 mitophagy protects against cytokine-induced neurotoxicity supports enhancing mitophagy in astrocytes as a promising therapeutic target.

Several intriguing findings warrant further investigation. Primarily, although inflammation can induce neurotoxic astrocytes, HK2-PINK1 activation promotes neuroprotection. In addition to metabolic functions, HK2 also participates in the innate immune response through interactions with mitochondrial antiviral signaling proteins. Whether HK2-PINK1-activated astrocytes represent a distinct reactive phenotype and how this pathway modulates inflammation remains unclear. This question is particularly relevant to PD, where loss of HK2-PINK1-activated astrocytes could exacerbate pathology by impairing mitophagy or reducing neuroprotective signaling. Beyond astrocytes, microglia also express HK2 and exhibit enhanced mitophagy. Understanding whether HK2-dependent mitophagy is unique to astrocytes will provide insight into glial interactions in disease. Previous studies have also proposed the concept of “transmitophagy,” whereby neurons shuttle damaged mitochondria to astrocytes for degradation. It remains to be determined whether the faster, more widespread mitophagy in astrocytes contributes to neuronal mitochondrial homeostasis. Finally, as these findings were generated in primary cultures, further *in vivo* studies are needed to understand how HK2 couples metabolism and mitophagy during changing energy demands.

In summary, our study provides evidence for faster, less spatially restricted PINK1-dependent mitophagy in astrocytes compared to neurons. This astrocyte-specific response is driven by the selective expression of the glycolytic enzyme HK2, which couples mitophagy to metabolism. Furthermore, we show that this pathway protects neurons against neuroinflammation. Understanding the intricacies of HK2-PINK1 will provide further insight into astrocyte mitophagy and its role in disease and may reveal a novel therapeutic avenue for PD and other neurodegenerative diseases.

## Data Availability

No new data produced or reported.
